# Can peripheral perfusion index predict disease mortality in COVID-19 patients in the emergency department^☆^

**DOI:** 10.1016/j.heliyon.2024.e35383

**Published:** 2024-07-30

**Authors:** Mehmet Gokhan Kaya, Ahmet Demir, Mehmet Reha Yilmaz, Kivanc Karaman

**Affiliations:** a Emergency Medicine Service, Yatagan State Hospital, Yatagan, Mugla, Turkey; b Department of Emergency Medicine, Faculty of Medicine, Mugla Sitki Koçman University, Mugla, Turkey

**Keywords:** COVID-19, Peripheral perfusion index, In-hospital mortality, Emergency department

## Abstract

**Background:**

Coronavirus disease 2019 (COVID-19) pneumonia remains a major public health concern. The prognostic efficacy of Peripheral Perfusion Index (PPI) has been researched in different pathologies such as trauma and sepsis. We hypothesized that PPI may serve as predictor of mortality in hospitalized patients with COVID-19 infection. This study aimed to describe the association between PPI at admission and COVID-19 mortality, a new mortality prediction tool.

**Methods:**

This retrospective, observational study was conducted at a tertiary care center in Turkey. Adult patients diagnosed with COVID-19 infection were enrolled in this study between Februrary 15, 2022 to April 15, 2023. Patient demographic and clinical data including vital signs, laboratory parameters and PPI on admission were collected from an electronic database. PPI was measured using Philips G30E patient monitor system. The primary outcome was in-hospital mortality.

**Results:**

In total, 200 patients with COVID-19 infection were included and 42 (21 %) in-hospital deaths were identified. For all parameters of study, age, oxygen saturation, respiratory rate, PPI, urea, creatinine, White Blood Cell (WBC), and High-sensitive cardiac Troponin T (hs-cTnT) values were significantly different between survivors vs non-survivors. hs-cTnT >21,25 pg/mL[HR:2.823 (95 % CI:1.211–6583)], PPI <2,15 [HR:2485 (95 % CI:1.194–5.175)], Oxygen saturation <87 % [HR:2258 (95 % CI:1.191–4.282)], and WBC >9680 x10^3^/ml [HR:2.124 (95 % CI:1.083–4.163)] were independent predictors of in-hospital mortality.

**Conclusions:**

This study identified the factors affecting in-hospital mortality among COVID-19 patients. Importantly, besides many parameter, PPI at admission was significantly associated with COVID-19 mortality and could be a feasible marker in emergency department to identify high risk patients.

## 1 Introduction

Coronavirus disease 2019 (COVID-19), caused by severe acute respiratory syndrome coronavirus 2 (SARS-CoV-2), is associated with substantial morbidity and mortality. As of November 19, 2023, over 772 million confirmed cases and over six million deaths have been reported globally [1]. Even though the World Health Organization has declared the pandemic to be over, the infection still persists, with reported increases in case and Intensive Care Unit (ICU) admission numbers, particularly in America and Europe [1]. Predicting undesirable outcomes is therefore crucial, even on admission to an Emergency Department (ED). In this context, urgent risk-stratification in the ED can lead to significantly reduced risks and better patient outcomes.

The extreme inflammatory response that occurs in severe cases of COVID-19, also known as a cytokine storm, causes worst outcomes such as sepsis, shock, or even death [2]. In this context, it is vital to detect this inflammatory response, which occurs in severe COVID-19 cases, at an early stage. For this purpose, many risk-stratification tools use clinical, physiological, and radiologic parameters have been investigated for prognostic effectiveness in COVID-19 cases [3,4]. Although risk stratification tools such as Shock Index, Diastolic Shock Index, Age Shock Index, Modified Shock Index are widely accepted and used markers for mortality, there is a need for new prognostic markers [5].

In this respect, Peripheral Perfusion Index (PPI) has emerged as a promising prognostic marker, particularly in septic patients, due to its characteristics of demonstrating changes in peripheral perfusion in the early stages, being noninvasive, objective, and reproducible [6]. The PPI is a valuable marker for assessing peripheral perfusion. PPI measures the ratio of the pulsatile to non-pulsatile components in peripheral blood flow and is primarily influenced by two key factors: cardiac output and the balance between the sympathetic and parasympathetic nervous systems. PPI tends to decrease in conditions of sympathetic dominance and/or reduced cardiac output, making it a reliable predictor of patient outcomes [7]. It has been suggested that PPI could be used almost as a vital sign even in the pre-COVID-19 era [8]. However, there is limited data in the literature regarding the use of such a valuable parameter in COVID-19 cases [9,10].

This study aimed to investigate the role of the PPI value assessed in the ED in predicting mortality in hospitalized COVID-19 cases.

## 2 Methods

### 2.1 Study design and settings

This retrospective, single-center, cross sectional, and observational study performed at the ED of university-affiliated training and research hospital in Muğla, Turkey. The hospital, comprising over 500 beds, registers an annual influx of approximately 100,000 adult visits to the ED. Ethical approval for the study was obtained from the local ethics committee (Reference Number: 230007–43). This study was conducted according to the Principles of the Declaration of Helsinki of 1975, as revised in 1983 and the Strengthening the Reporting of Observational Studies in Epidemiology (http://www.strobe-statement.org) guidelines. The need to obtain written consent informs was waived because of the retrospective nature of the study.

### 2.2 Patients selection

From Feb 15, 2022 to Apr 15, 2023, all consecutive adult patients (aged ≥18 years old) admitted to ED and hospitalized with laboratory-confirmed COVID-19 infection based on positive Reverse-Transcriptase-Polymerase Chain-Reaction (RT-PCR) assay via oropharyngeal or nasopharyngeal swabs, were enrolled. However, certain groups were excluded to maintain the study's integrity. Patients, who had a history of peripheral artery disease, who had sought hospital discharge against medical advice, who had missing data in medical records and who had been referred to other hospitals were excluded. In addition, patients requiring acute interventional procedures and/or with specific diagnoses (acute myocardial infarction, acute aortic syndrome, pulmonary embolism, acute appendicitis, etc.) were excluded from the study, because they were not admitted to the hospital due to COVID-19 infection, even if they were incidentally found to have a positive COVID-19 test.

### 2.3 Data collection

Clinical data included demographic characteristics (age and gender), vital signs (systolic and diastolic blood pressure, heart rate, respiratory rate, fever, and oxygen saturation), PPI, and laboratory parameters at ED admission including complete blood count, renal and liver function tests, serum electrolytes, and High-Sensitive Cardiac Troponin T (hs-cTnT) levels, were extracted from medical records and recorded.

All patients admitted to ED with COVID-19 infection symptoms were evaluated in the COVID-19 area of ED and a trained triage nurse (always available in ED), who was unaware of the study, took vital sign and PPI measurements. The PPI is a new parameter developed for hemodynamic monitoring, derived from the Photoplethysmography (PPG) method, which is traditionally used for pulse oximetry monitoring. PPI is derived from PPG signal and represents the ratio of pulsatile on non-pulsatile light absorbance or reflectance of the PPG signal. PPI determinants are complex and interlinked, involving and reflecting the interaction between peripheral and central haemodynamic characteristics, such as vascular tone and stroke volume. Philips G30E monitor system automatically calculates PPI via pulse oximetry probe and displays it on the screen, thanks to the software it contains.

### 2.4 Outcome measures

The outcome of present study is to determine the prediction power of PPI on in-hospital mortality among hospitalized COVID-19 patients.

### 2.5 Statistical analysis

IBM SPSS v.25.0 package program (SPSS Inc., Chicago, IL) was used to analyze the recorded data. Normality analyses of the data were conducted using the Kolmogorov–Smirnov test. Because of non-normally distribution, all quantitative data were presented as median (minimum-maximum). The categorical variables were expressed as the number of cases (n) with percentages (%). The differences between the groups were investigated using the Mann–Whitney *U* test. Intragroup comparisons of the categorical variables were made using the chi square test and the Fisher's exact test. Receiver operating characteristic (ROC) analysis was performed to determine the in-hospital mortality predictive power of the PPI and other significantly differiantiated parameters. The optimum cut-off levels were determined using Youden's index (sensitivity + specificity ─ 1). The sensitivity, specificity, and positive and negative predictive values were calculated for the optimum cut-off levels. Univariate and Multivariate Cox proportional hazard models were fitted to study the association between the distributions of risk factors among survival. The results of the analysis were presented in terms of the estimated hazard ratios (HR) with 95 % confidence intervals (95 % CI). Statistical significance was set at p < 0.05.

## 3 Results

**Fig. 1:**
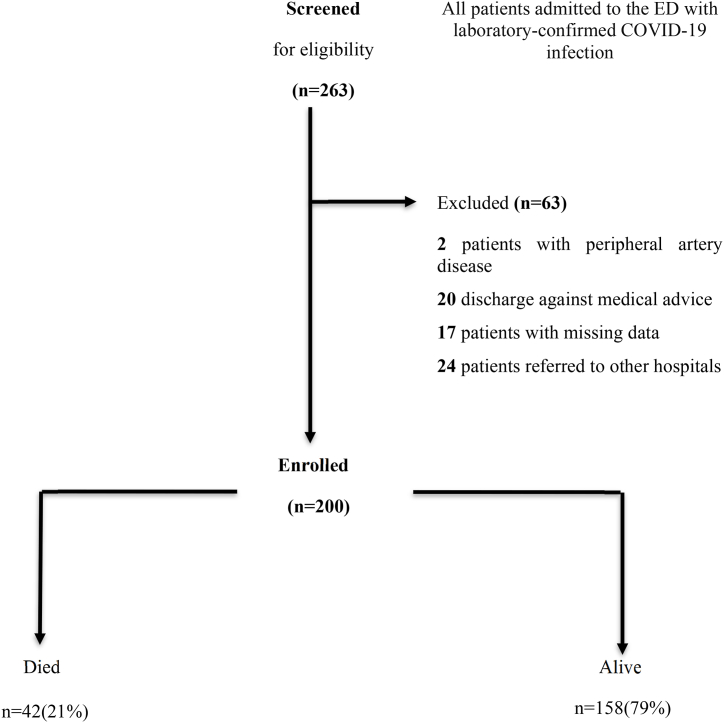
Flow diagram of study cohort.

A total of 263 patients who were admitted to ED and hospitalized with RT-PCR confirmed COVID-19 infection from Feb 15, 2022 to Apr 15, 2023 were identified. Of these 263 patients, 63 excluded. The remaining 200 patients were included (Fig. 1).

Of these 200 patients, 115 (%57,5) were male, and median age was 74 (20–97) years. The median PPI was 2,40 (0,50–9,30), and median lenght of stay was 7 (1–100) days. Of the patients included in the study, 42 (21 %) died during their hospital stay. A summary of demographic, laboratory and clinical data are presented in Table 1.

**Table 1:** The summary of demographic, laboratory and clinical data of patients.

Variable		All patients (n = 200)	Survivors (n = 158)	Non-survivors (n = 42)	P value^a^
*Age (years), median (min-max)*		74 (20–97)	72 (20–97)	80,5 (42–92)	0,010
*Gender, n(%)*	*Male*	115 (57,5)	29 (69,0)	86 (54,4)	0,089
	*Female*	85 (42,5)	13 (31,0)	72 (45,6)	
*Vital signs*	*Systolic BP (mmHg)*	133 (70–237)	133 (82–237)	133 (70–204)	0,452
	*Diastolic BP (mmHg)*	75,5 (37–127)	76 (37–127)	75 (40–114)	0,901
	*Pulse (per minute)*	93 (40–157)	92,5 (42–157)	94,5 (40–155)	0,461
	*Respiratory rate (per minute)*	20 (12–36)	20 (12–36)	26 (12–36)	**<0,001**
	*Saturation (%)*	92 (45–100)	93 (50–100)	85 (45–100)	**<0,001**
	*Fever (°C)*	36,5 (35,1–40,2)	36,5 (35,1–40,2)	36,5 (36,0–38,5)	0,634
*Peripheral Perfusion Index (%)*		2,40 (0,50–9,30)	2,80 (0,50–9,30)	1,50 (0,50–6,20)	**<0,001**
*Hospital stay (day)*		7 (1–100)	6,5 (1–100)	8 (1–81)	0,366
*Laboratory Parameters*	*Urea*	45,2 (17,0–225,0)	40,2 (17,0–195,0)	77,2 (20,3–225,0)	**<0,001**
	*Creatinine*	1,03 (0,55–8,50)	0,97 (0,55–8,50)	1,38 (0,64–7,24)	**<0,001**
	*Sodium*	134 (110–163)	134 (110–163)	132,5 (117–145)	0,183
	*Potassium*	4,3 (0,8–6,4)	4,2 (0,8–6,4)	4,3 (2,6-6,0)	0,294
	*AST*	25,1 (5,0–420,0)	24,1 (5,0–380,0)	32,0 (10,0–420,0)	0,062
	*ALT*	16,1 (5,0–583,0)	15,7 (5,0–583,0)	17,1 (5,0–564,0)	0,566
	*WBC*	8,06 (1,27–33,50)	7,53 (1,85–33,50)	10,65 (1,27–28,19)	**0,005**
	*Hgb*	11,9 (5,3–17,4)	11,9 (5,3–17,0)	11,7 (6,9–17,4)	0,777
	*Platelet*	212,5 (12–568)	212,5 (12–568)	215,5 (32–568)	0,888
	Hs-cTnT	14,52 (3,0–1143,0)	11,8 (3,0–625,9)	37,5 (5,2–1143,0)	**<0,001**

ALT: Alanine aminotransferase, AST: Aspartate aminotransferase, BP: Blood Pressure, Hgb: Haemoglobin, Hs-cTnT: High-sensitivity cardiac troponin T test, Max: Maximum, Min: Minimum, WBC: White Blood Cell.

a Group comparisons were made between survivors vs non-survivors.

**Fig. 2:**
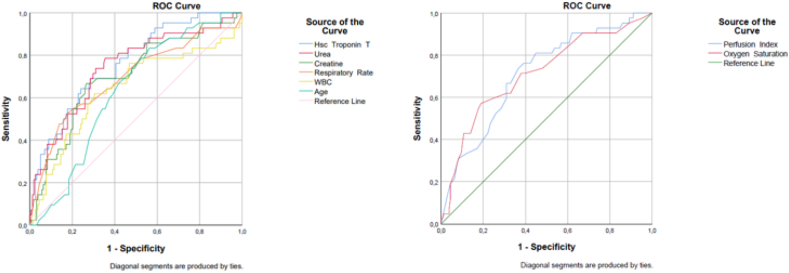
*ROC Analysis Showing the Relationship Between Survivors vs Non-survivors*. Receiver operating characteristic curves for the hs-cTnT, Urea, Creatine, Respiratory Rate, WBC, and Age (**A**); the Peripheral Perfusion Index, and Oxygen Saturation (**B**) predicting in-hospital mortality in COVID-19 patients. * hs-cTnT: High-sensitivity cardiac troponin T test, WBC: White Blood Cell.

For all parameters of study, age, oxygen saturation, respiratory rate, PPI, urea, creatinine, White Blood Cell (WBC), and hs-cTnT values were significantly different between survivors vs non-survivors. We performed ROC analysis to determine the in-hospital mortality predictive power of significantly different parameters mentioned before. The highest AUC value was belong to hs-cTnT, while the lowest was belong to age. Table 2 and Fig. 2 presents the results of ROC analysis in detail.

**Table 2:** Diagnostic Effectiveness of Parameters in the Differentiation of Between Survivors vs Non-survivors.

	AUC (95 % CI)	Sensitivity (%)	Specificity (%)	PPV (%)	NPV (%)	*p* value
Hs-cTnT >21,25 pg/mL	**0,761** (0,683–0,840)	69,0	71,5	39,2	89,7	**<0,001**
Urea >50,0 mg/dl	0,740 (0,653–0,828)	78,6	64,6	37,1	**91,9**	**<0,001**
PPI <2,15 %	0,712 (0,626–0,798)	73,8	63,3	34,8	90,1	**<0,001**
Oxygen Saturation <87 %	0,712 (0,620–0,804)	57,1	81,0	**44,4**	87,7	**<0,001**
Creatinine >1,25 mg/dl	0,700 (0,609–0,792)	66,7	73,4	40,0	89,2	**<0,001**
Respiratory Rate >25 breath/min	0,692 (0,594–0,790)	52,4	**82,3**	44,0	86,7	**<0,001**
WBC >9680 x10^3^/ml	0,640 (0,536–0,744)	61,9	69,6	35,1	87,3	**0,005**
Age >68 years	0,630 (0,546–0,714)	**83,3**	43,7	28,2	90,8	**0,010**

* hs-cTnT: High-sensitivity cardiac troponin T test, PPI: Peripheral Perfusion Index WBC: White Blood Cell, PPV: Positive predictive value NPV: negative predictive value. CI: Confidence Interval AUC: Area Under Curve.

Univariate Cox regression analysis, showed that oxygen saturation, respiratory rate, PPI, urea, creatinine, WBC, and hs-cTnT were significant factors for in-hospital mortality (Table 3). Consequently, independent prognostic factors associated with in-hospital mortality were figured out by multivariate Cox regression analysis. The independent predictors related to in-hospital mortality were as follows: hs-cTnT [>21,25 pg/mL: HR = 2.823 (95 % confidential interval (CI) = 1.211–6583), p: 0.016], PPI [<2,15: HR = 2485 (95 % CI = 1.194–5.175) p: 0.015], oxygen saturation [<87 %: HR = 2258 (95 % CI = 1.191–4.282) p: 0.013], WBC [>9680 x10^3^/ml: HR = 2.124 (95 % CI = 1.083–4.163) p: 0.028]. The detailed information was shown in Table 3.

**Table 3:** Univarite and multivarite Cox regression results.

	Univariate Cox Regression Analysis		Multivariate Cox Regression Analysis	
	HR (95 % CI)	p value	HR (95 % CI)	p value
hs-cTnT >21,25 pg/mL	3966 (2042–7702)	**<0,001**	2823 (1211–6583)	**0,016**
Urea >50,0 mg/dl	4117 (1962–8640)	**<0,001**	2652 (0,894–7870)	0,079
PPI <2,15 %	3377 (1690–6749)	**0,001**	2485 (1194–5175)	**0,015**
Oxygen Saturation <87 %	2332 (1243–4375)	**0,008**	2258 (1191–4282)	**0,013**
Creatinine >1,25 mg/dl	3067 (1603–5871)	**0,001**	0,511 (0,170–1537)	0,232
Respiratory Rate >25 breath/min	2334 (1260–4324)	**0,007**	1675 (0,834–3365)	0,147
WBC >9680 x10^3^/ml	2984 (1586–5616)	**0,001**	2124 (1083–4163)	**0,028**
Age >68 years	0,469 (0,205–1073)	0,073	–	–

* hs-cTnT: High-sensitivity cardiac troponin T test, PPI: Peripheral Perfusion Index WBC: White Blood Cell.

## 4 Discussion

Our study focused on the utility of PPI as a predictive parameter of mortality in patients hospitalized with COVID-19 in ED. We found that lower PPI values is associated with increased risk of mortality. A cutoff PPI value of 2,15 predicted mortality with a sensitivity of 73,8 % and a specificity of 63,3 %. The PPI, as a noninvasive, easily measurable, subjective/quantitative parameter of peripheral perfusion, has previously been investigated as a prognostic marker in the evaluation of many diseases in the ED [7]. Although it has been demonstrated to be valuable in predicting both the severity of the disease and cardiovascular complications in COVID-19 patients, the majority of these studies have been conducted in intensive care and/or in patient units. To the best of our knowledge, this is the first study that demonstrated the successful utilization of PPI in predicting mortality among COVID-19 patients in the ED.

Considering the critical need for early identification, prompt intervention, and accurate risk assessment, it becomes paramount for emergency physicians to distinguish patients at high mortality risk among those severely afflicted with COVID-19. Hence, it's not unexpected to witness a surge in research dedicated to developing straightforward and convenient prediction tools. These tools aim to aid emergency physicians in swiftly and accurately identifying patients at elevated risk of mortality. For this reason, it is not surprising to see a growing number of studies focusing on simple and convenient prediction tools that can help emergency care physicians to more rapidly and effectively recognize patients at high risk of mortality. In a recent study from Turkey published by Korkut et al. PPI is an easy to apply and useful parameter in the ED in determening the severity of COVID-19 patients [11]. Another study conducted by Akdur et al., in our country, which has methodological similarities to our study, demonstrated that low PPI (<1.5) values are associated with mortality in COVID-19 patients. Although the patient populations in both studies are similar, there are differences in the study parameters and outcomes. While Akdur and colleagues examined parameters such as NEWS score, Shock Index, and Computed Tomography-Severity Score in addition to PPI, our study focused specifically on PPI. Additionally, while their study examines 14- and 90-day mortality as outcomes, our study focuses on in-hospital mortality [4]. Although the investigated time-to-death and the resulting cut-off values may differ, the findings of our study are consistent with the literature. A decreased PPI value (<2,15) demonstrated a significant association with in-hospital mortality (HR 2.485; 95 % CI 1.194–5.175; *p: 0.015*). In the prognostic evaluation of COVID-19 patients in ED, PPI appears to be as effective as many known parameters such as age, respiratory rate, oxygen saturation, kidney function tests and hs-cTnT.

Our study focused on the impact of PPI on mortality; however, vital signs, laboratory parameters, and demographic data are also part of our research. Recent findings have underscored the significance of several biochemical tests, either independently or in combination, in predicting COVID-19-related mortality. In a meta-regression analysis published by Zhang JJY et al., observed that higher leukocyte counts, elevated levels of ALT, AST, lactate dehydrogenase (LDH) and raised procalcitonin levels were note-worthy predictors of admission to intensive care unit. Further, the researchers found that elevated LDH and high leukocyte counts were significantly associated with COVID-19 led mortality [12]. In a comprehensive meta-analysis conducted by Izcovich et al., similar to our study, 49 parameters such as increasing age, elevated myocardial injury markers, increased WBC, rising plasma creatinine, and blood urea nitrogen levels were identified as high/moderate predictors of poor prognosis [[13], [a]]. In parallel to these results in our study, elevated hs-cTnT levels, increased WBC counts, and raised plasma creatinine and urea levels were significantly different in deceased patients. Furthermore, a higher hs-cTnT and WBC values demonstrated a significant association with in-hospital mortality (HR 2.823, 95 % CI 1.211–6.583; HR 2.124, 95 % CI 1.083–4.163; respectively). In previous studies, vital signs were reported to be associated with mortality in patients with COVID-19. A clinical study from US reported that heart rate (higher), respiratory rate (higher), and oxygen saturation (lower) were associated with COVID-19 mortality [14]. Another study from Turkey also demonsrated that low levels of oxygen saturation was associated with 30-day mortality (OR 0,69; p:0.052) [15]. Similarly to the literature, our study also found that low levels of oxygen saturation were associated with in-hospital mortality. (HR 2.258, 95 % CI 1.191–4.282, *p:0.013*).

Our study has some limitations. First, this was a singlecenter, retrospective study with a small sample size, and therefore, the findings of the study cannot be generalized. Second, we only focused on patients with hospitalized COVID-19 patients; COVID-19 patients discharged from ED were not analyzed. Third, we did not assess medications that affect vital signs such as beta-blockers, and arrhythmias and co-morbidities that affect mortality were not identified. Fourth, vital signs were measured at a single time point, which may not accurately reflect disease dynamics. Finally, before its application in clinical practice, the PPI should undergo external validation in a large-scale studies.

## 5 Conclusions

This study demonstrated that the initial PPI is a significant prognostic factor in patients with COVID-19 infection. In clinical settings it may contribute to patient triage as much succesfully as traditional vital signs and laboratory parameters. PPI can be easily used and can be applied for risk stratification of in hospital mortality of in COVID-19 patients.

## Funding

This research received no external funding.

## Data availability statement

Data available on request.

## CRediT authorship contribution statement

**Mehmet Gokhan Kaya:** Writing – original draft, Resources, Methodology, Data curation, Conceptualization. **Ahmet Demir:** Writing – original draft, Formal analysis. **Mehmet Reha Yilmaz:** Writing – review & editing. **Kivanc Karaman:** Writing – review & editing, Writing – original draft.

## Declaration of competing interest

None.

## References

[1] (2021). World health organization WHO coronavirus 2019 (COVID-19) pandemic. https://www.who.int/emergencies/diseases/novel-coronavirus-2019.

[2] Fajgenbaum D.C., June C.H. (2020). Cytokine storm. N. Engl. J. Med..

[3] Incerti D., Rizzo S., Li X. (2021). Prognostic model to identify and quantify risk factors for mortality among hospitalised patients with COVID-19 in the USA. BMJ Open.

[4] Akdur G., Daş M., Bardakci O. (2021). Prediction of mortality in COVID-19 through combing CT severity score with NEWS, qSOFA, or peripheral perfusion index. Am. J. Emerg. Med..

[5] Avci M., Doganay F. (2022). Prognostic performance of shock index, diastolic shock index, age shock index, and modified shock index in COVID-19 pneumonia. Disaster Med. Public Health Prep..

[6] Lima A., Jansen T.C., van Bommel J., Ince C., Bakker J. (2009). The prognostic value of the subjective assessment of peripheral perfusion in critically ill patients. Crit. Care Med..

[7] Oskay A., Eray O., Dinç S.E., Aydın A.G., Eken C. (2015). Prognosis of Critically ill patients in the ED and value of perfusion index measurement: a cross-sectional study. Am. J. Emerg. Med..

[8] Elshal M.M., Hasanin A.M., Mostafa M., Gamal R.M. (2021). Plethysmographic peripheral perfusion index: could it Be a new vital sign?. Front. Med..

[9] de Souza G.M., Galindo V.B., Rocha D.L. (2022). Assessment of peripheral perfusion in severe acute respiratory syndrome coronavirus 2 (Sars-cov-2) infection: an exploratory analysis with near-infrared spectroscopy. Research Square.

[10] Menon A., Divya R. (2022). Use of peripheral perfusion index (PI) as a predictor of cardiovascular complications in hospitalized Covid 19 patients - a pilot study. Biomedicine.

[11] Korkut M., Bedel C., Selvi F., Zortuk Ö. (2022). Can peripheral perfusion index (PPI) predict disease severity in COVID-19 patients in the emergency department?. Ibnosina J. Med. Biomed. Sci..

[12] Zhang J.J.Y., Lee K.S., Ang L.W., Leo Y.S., Young B.E. (2020). Risk factors for severe disease and efficacy of treatment in patients infected with COVID-19: a systematic review, meta-analysis, and meta-regression analysis. Clin. Infect. Dis..

[13] Izcovich A., Ragusa M.A., Tortosa F. (2020). Prognostic factors for severity and mortality in patients infected with COVID-19: a systematic review. PLoS One.

[14] Rechtman E., Curtin P., Navarro E., Nirenberg S., Horton M.K. (2020). Vital signs assessed in initial clinical encounters predict COVID-19 mortality in an NYC hospital system. Sci. Rep..

[15] Satici M.O., Islam M.M., Satici C. (2022). The role of a noninvasive index 'Spo 2/Fio2' in predicting mortality among patients with COVID-19 pneumonia. Am. J. Emerg. Med..

